# Our Correspondence

**Published:** 1872-01

**Authors:** 


### Our Correspondence.

DR. B.—*Jersey City.*—Cunderango is a humbug. We do not deal in it.

D. M.—*Hamilton, O.*—We can procure you cataract glasses of the number you require.

DR. P.—*Wellsboro, Pa.*—"*Good Health*," published by Alex. Moore, Boston, is perhaps, the best edited, popular, health journal published.

A. R.—*Hannibal, Mo.*—Consult back numbers of BISTOURY, Domestic Medicine Dept., for recipe for worms in children.

MISSES O. B. and J. A. H.—*Coalmount, Pa.*, are informed that we do not supply sample copies of journals with which we club.

DR. O.—*Poughkeepsie, N. Y.*—Lallemand's porte caustique is what you need. Can be procured of Geo. Tiemann & Co., N. Y.

DR. B.—*Vincennes, Ind.*—Geo. Tiemann & Co., will send you directions and diagrams for measurements of elastic stockings. They have an Agency in Chicago, we believe.

MRS. T. B. S.—*Kiesville, Ky.*—Consult some competent surgeon near you. The traveling Quack is trying to deceive you. Do not trust him.

T.—*Harrisburg, Pa.*—Your symptons are those of stone. Apply to some surgeon and have your bladder sounded, and cease taking nostrums.

P.—*Washington, D. C.*—That is out of our line. No interferance is necessary—a better physician will remedy her difficulty in the course of nine months. You should be ashamed of yourself for contemplating such an act, and should be exposed.

MRS. S.—*Williamsport, Pa.*—Do not meddle with your ears. They do not require "cleaning out." The cerumen is placed there "on purpose,"—let it alone. Apply two or three drops of pure glycerine, occasionally, confining it in the ear, over night, by means of a pleget o cotton.

J. R. L.—*Binghamton, N. Y.*—There is nothing the matter with the refractive power of your eye, therefore no glasses are required. If you would consult an oculist instead of a jeweler, or dealer in glasses, perhaps your impaired vision might be restored.

---

DR. S. R.—*Oswego, N. Y.*—The supporter you refer to, we know nothing of save that its description resembles too closely a first-class Hoe Printing Press. It is altogether too complicated to be useful. "Dr. Babcock's Silver Uterine Supporter," is by far the best that has yet been invented. Having employed it in our practice for several years, we can safely and heartily recommend it.

---

PENN YAN, N. Y., Oct. 20, 1871.

DR. UP DEGRAFF:

Will you be kind enough to inform me whether Dr. —— the traveling oculist,is reliable or not? S. O. M.

—As reliable as *any* "traveling doctor" can be. You must be "hard up" for Physicians in Penn Yan,if you have to seek advice from *any* traveling doctor.

---

LOUISVILLE, Ky., Oct. 12, 1871.

DR. UP DEGRAFF:

*Dear Sir:*—I saw your BISTOURY at the office of a physician and thought I'd write you for information concerning Hoyt's work on the generative organs. Is it reliable, and would you accept the advice therein contained. I see you advise your readers in such matters, therefore am ecouraged to write you. Please reply by mail, and oblige. S——.

You did not enclose a stamp for a "reply by mail," so will answer you here. Three cents to one individual, occasionally, does not amount to much, but when from three to five hundred write us every quarter, for information upon some point, asking us to "please reply by mail, and not through the BISTOURY," without enclosing a postage stamp for such reply, we must respectfully decline all such engagements—they are altogether *too* free.

The book you speak of is an advertisement for a quack in New York, who expects you to employ him to treat your case. While he plays upon your fears, by attempting to describe your symptoms and picturing their fearful results, he is careful to say nothing of the means of cure, save that which is supposed to be vested in his own hands. Such men are not to be trusted.

PITTSBURGH, Pa., Nov. 5, 1871.

THAD S. UP DE GRAFF, M. D.

*Dear Sir:*—Here is a precious document sent me from a Quack manufacturing machine in Philadelphia. I send you the letter for publication in the BISTOURY, in order that the people may judge of the merits of a graduate of a school, one of the Professors (?) of which would be guilty of writing such a villainous letter.—You have my authority for publishing the letter in full and giving my name if you so desire.

Yours Truly,

—— —— M. D.

COLLEGE BUILDING, 514 Pine-St.,<br>Phila., Nov. 1st, 1871.

*Dear Sir:*—Do you know of any one who wishes to obtain a diploma of the Pennsylvania University, the oldest Medical College in the U. S. If so, I know of a party that will dispose of his, and he wishes me to find him a customer for it. The price is $350. His name can be taken out and another put in so that it never can be detected. The date can be fixed in the same manner, no one know the difference. This matter is confidential and on the square; the party is hard up for cash which induces him to dispose of it. I have known of a number of parties that have disposed of their diplomas in the same way. Should you know of a party that wants something of the kind, let me know and I can obtain it for them. This is a matter that I wish kept on the square, and as a Masonic secret as well as a professional secret.

Fraternally Yours,

A. P. BISSELL, M. D., ⊏⊐<br>914 Lombard-St.

—The same Dr. Bissell was traveling through this part of the country last summer conferring his miserable degrees (?) upon all so-called doctors who were in need of such a document. Upon inquiry, we learn that the Institution is really chartered by the State Legislature, and that the College (?) has been doing just this kind of business for years. Is there no way of suppressing the concern? It is a disgrace to the city that harbors it. Why don't the medical fraternity of Philadelphia apply to the Legislature for relief from such a nuisance? We suppose that *square* with which the Doctor or Professor ornaments his name,is intended to convey the idea that he is a Mason. We don't believe it,—if he is, kick him out!

